# Detection of Vancomycin Resistant Enterococci from Rectal Swab Samples by Becton-Dickinson GeneOhm VanR assay and Culture at ICU of a Tertiary Care Center in Turkey

**DOI:** 10.12669/pjms.292.2567

**Published:** 2013-04

**Authors:** Ayhan Gozaydin, Sukran Kose, Gulfem Ece, Gursel Ersan, Mustafa Gonullu

**Affiliations:** 1 Ayhan Gozaydin, Infectious Diseases and Clinical Microbiology Specialist, Department of Infectious Diseases and Clinical Microbiology, Tepecik Education and Research Hospital, Izmir, Turkey.; 2 Sukran Kose, Infectious Diseases and Clinical Microbiology Specialist, Department of Infectious Diseases and Clinical Microbiology, Tepecik Education and Research Hospital, Izmir, Turkey.; 3 Gulfem Ece, Clinical Microbiology Specialist, Department of Infectious Diseases and Clinical Microbiology, Tepecik Education and Research Hospital, Izmir, Turkey.; 4 Gursel Ersan, Infectious Diseases and Clinical Microbiology Specialist, Department of Infectious Diseases and Clinical Microbiology, Tepecik Education and Research Hospital, Izmir, Turkey.; 5 Mustafa Gonullu, Anesthesiology and Reanimation Specialist, Department of Anesthesiology and Reanimation, Tepecik Education and Research Hospital, Izmir, Turkey.

**Keywords:** Conventional methods, *Enterococci*, Molecular methods, Vancomycin

## Abstract

***Objectives: ***Vancomycin resistance is due to change in ligase enzyme that destroys the binding of the drug. The gold standard is culture; but now molecular methods have also been developed. The aim was to detect the VRE rate at ICUs by culture and BD GeneOhm™ VanR and compare the results of both assays.

***Methodology:*** 135 perianal swabs were taken from the patients at ICUs between January 1^st^ 2009 and April 30^th^ 2009. Samples were identified by conventional methods and BD GeneOhm VanR assay.

***Results:*** In newborn ICU, 41 patients (74.6%) were negative by both methods. Two (3.6%) were positive by both methods. Twelve (21.8%) of them were culture negative and PCR positive. In adult ICU, 73 (91.3%) patients were negative by both methods. Seven patients (8.8%) were positive by molecular method only.

***Conclusion:*** This study showed low VRE positivity due to factors like inhibition in PCR or culture negativity due low inoculum for bacterial growth. Early detection of VRE is an important issue especially in ICUs and molecular techniques are important tools; but against all, we still need to confirm this method with culture based techniques and in order to do this further studies with higher number of patients with VRE colonisation are required.

## INTRODUCTION

Enterococci which are Gram positive cocci in chain forming can lead to nosocomial and community-acquired infections.^1^ They are a part of colon flora and can grow in 6.5% NaCl containing blood agar medium.^2^ Vancomycin is an important treatment choice for enterococci.^3^ Vancomycin-resistant enterococcus (VRE) became one of the most important causes of nosocomial infection after being first identified in 1986.^4^ The most common method to detect VRE isolates is the culture based methods that take 1-5 days.^5^^-^^7^

Recently rapid molecular diagnostic methods were also reported for VRE screening.^7^ The BD GeneOhm VanR assay (BD GeneOhm, San Diego, CA) is a U.S. Food and Drug Administration approved test in vitro test for VRE screening directly from perianal swabs. This rapid molecular method helps to detect VRE carriers in less amount of time compared to culture based methods which may take days to report the result and also provide the chance of avoiding the spread from one patient to another. The aim of our study was to detect the rate of vancomycin resistant enterococci isolated from perianal swab samples of the patients hospitalized at intensive care units of Tepecik Education and Research Hospital by culture and Becton-Dickinson GeneOhm™ VanR assay and compare the results of both assays.

## METHODOLOGY

Perianal swab samples were taken from the patients hospitalized at the intensive care units of Tepecik Education and Research Hospital between January 1^st^ 2009 and April 30^th^ 2009. Patients’ demographic data and underlying diseases were also recorded. Both samples for RT-PCR and culture methods were from the same (duplicated) perianal swabs. The identification was done by BBL CRYSTAL Gram-Positive (GP) identification (ID) system (Becton-Dickinson, USA). The antimicrobial susceptibility was studied by Kirby-Bauer disk diffusion method. Penicilin (10U), ampicilin/sulbactam (10μg/10μg), gentamycin (120μg), streptomycin (300μg), ciprofloxacin (5μg), levofloksasin (5μg), erythromycin (15μg), linezolid (30μg), vancomycin (30μg) and teikoplanin (30μg) antimicrobial disks (Becton-Dickinson, USA) were applied.^8^ The MIC values of vancomycin resistant isolates were studied by E-test (AB, Biodisk, Solna, Sweeden). The BD GeneOhm™ VanR Assay is a qualitative in vitro test for the rapid detection of vancomycin-resistance (*vanA* and *vanB*) genes directly from perianal swabs. The assay was performed and evaluated according to manufacturer's instructions.^9^

## Results

A total of 135 patients were included into the study, 71(52.6%) were male, 64 (47.4%) were female. There were 55 newborns and 80 adult patients. The demographic data and the underlying disorders are shown in Table-I. The risk factors of the patients at adult ICU and the newborn ICU were shown in Tables II and III. A total of 43 (31.8%) enterococci were isolated. They consisted of 38 *E. faecium*, two *E.* *faecalis,* one *E.* *durans*, one *E.* *solitarius* and one *E.* *avium*. *E.* *faecalis* isolates were 50% susceptible to penicilline, ampicilin-sulbactam, erithromycin, ciprofloxacin, levofloxacin, high level streptomycin and gentamycin; and 100% susceptible to vancomycin, teicoplanin and linezolide.

The antimicrobial suceptibility of *E.* *faecalis* isolates were 5.2% to penicillin, 5.2% to ampicilin-sulbactam, 13.1% to erithromycin, 28.9% to ciprofloxacin, 28.9% to levofloxacin 36.8% to high level streptomycin, 36.8% to high level gentamycin, 94.8% to vancomycin, 94.8% to teicoplanin, and 100% to linezolide. Only two *E. faecium* isolates (3.6%) isolated at newborn ICU were resistant to vancomycin and teicoplanin. Vancomycin MIC values for both isolates were >256μg/ml, and teikoplanin MIC values were >256μg/ml and 64 μg/ml repectively. Other than these two isolates, neither vancomycin nor teicoplanin resistance was detected. Molecular assay and culture results of the adult ICU and newborn ICU were shown in Table-IV.

**Table-I:** Demographic data of the patients

	*Adult ICU (N=80)*		*Newborn ICU (N=55)*	
*Age/Birth week*	*67.4*		*30.9 week*	
Gender	Male	38(47.5%)	Male	33(60%)
	Female	42(52.5%)	Female	22(40%)
Diagnosis	Pneumoniae	13 (16.2%)	Prematurity	39(70.9%)
	Cerebrovascular Disease	11(13.7%)	RDS	27(49.1%)
	More than one underlying disorder	25(31.2%)	More than one underlying disorder	37(67.3%)
Mean hospital stay (day)	9.6 (2–70)		24.1 (2–77)	
Antibiotic therapy	Cephaperasone/sulbactam	22.5%	Aminoglycoside	36.4%
	Metronidazole	16.2%	Carbapenem	25.4%
	Carbapenem	13.7%	Ampicilin/sulbactam	20%
	Cephasoline	10%	Teikoplanin	18.2%
	Linezolide	8.7%	Antifungal treatment	10.9%
	Vancomycin	1.2%	Vancomycin	0,00%
	Teikoplanin	2.5%	Teikoplanin	18.2%

**Table-II:** Risk factors of the patients hospitalized in the newborn ICU. Also the risk factors of PCR positive ones

*Risk factor*	*Patient number* *n=55*	POS NA by PCR* *n=14*
Renal failure	0	0
GIS Operation	1	0
Corticosteroid treatment	0	0
Invasive procedure	30	10
Enteral nutrition	27	10
Glycopeptide use	35	8

*vanA type resistance detected by PCR

**Table-III:** Risk factors of the patients hospitalized at adult ICU and the risk factors of the ones positive by PCR

* Risk factors*	*Patient number* *n=80*	POS NA by PCR* *n=2*	Presumptive POS NA by PCR ** *n=4*	Positive NA*** *n=1*
Renal failure	12	0	2	0
GIS Operation	11	0	0	0
Dibetes mellitus	32	1	3	0
Malignity	6	1	0	0
Corticosteroid treatment	15	1	1	0
Invasive procedure	54	1	3	1
High APACHE II score	54	2	3	1
Previous hospitalization	10	0	0	1
Enteral nutrition	45	2	3	1
Glycopeptide use	60	2	3	0

*vanA type resistance detected, **vanB type resistance detected, *** DNA of *vanA* and* vanB* type resistance detected.

**Table-IV:** Molecular assay and culture results of the adult ICU and newborn ICU.

*N-ICU ((n=55)*	*Pt No (%)*	*Isolates and van gene*
C neg/PCR neg	41(74.6)	
C pos/PCR pos	2(3.6)	*E. faecium* 2 (*vanA* 2)
C neg/PCR pos	12(21.8)	*E.faecium* 12(*vanA* 12)
Total	55(100)	
A-ICU (n=80)	Pt No(%)	Isolates and *van *gene
C neg/PCR neg	73(91.3)	
C pos/PCR pos	0(0)	
C neg/PCR pos	7(8.8)	*E.faecium* 7( *vanA* 2, *vanB* 4, both *vanA *and *vanB *1)
Total	80(100)	

Notes: C: culture, PCR: GeneOhm TM, N-ICU: Newborn ICU, A-ICU: Adult ICU

## DISCUSSION

The first isolates of VRE were reported at the end of 1980s. After this VRE infection and colonisation showed an increase.^10^ The resistance is troublesome for the management of hospitalized patients. Preventing the spread of VRE is an important matter.^11^ The detection of VRE is based on culture, which requires 24 to 72 hours.^12^ CLSI recommends the use of 6 µg/ml vancomycin containing agar.^8^ The first VRE isolate in Turkey was detected in 1998 at Akdeniz University Hospital and the reports followed there after.^13^ Tufan ZK et al reported a study on screening of VRE carriers in total of 508 intensive-care unit patients and health care staff. Risk factors such as previous antibiotic use, especially vancomycin and cephalosporin, the presence of invasive devices like catheters, and co-morbid diseases were investigated. Rectal smear cultures were obtained from each participant to detect VRE colonization. Except for one patient, who had been transferred from another hospital, no VRE colonization was detected in patients or health care staff.^14^

Yis R et al evaluated the status of VRE colonization in Oncology Department of Gaziantep Children’s Hospital following a VRE isolation from the urine sample of a patient.^15^ Perirectal swab samples were collected from patients. A total of 123 perirectal swab specimens obtained from patients staying at oncology, burn, pediatric surgery and ICUs were investigated and the rate of VRE colonization was 14.6%. In our study the rate of VRE positivity was less than this one; because of the fact that our study was not conducted during an outbreak as stated above and in the study above they did not apply a molecular method as well which could have detected the VRE positivity earlier.^15^

Zer Y et al investigated the molecular epidemiology and antibacterial resistance of *Enterococci* strains isolated from in-patients of the 800-bed capacity research hospital of the Faculty of Medicine, University of Gaziantep between January 2009 and August 2010. *vanA* gene-type resistance was recorded in 76 (93.8%) of the strains, *vanB* gene-type resistance in two (2.5%) and nonA-nonB type resistance gene in three (3.7%) of the strains.^16^ In our study we had only two positive patients by both methods and had less *van A *and *van B *positivity and this may be due to inhibition in molecular method or strict application of infection control measures. Most of the studies that we considered in Turkey are about the evaluation of outbreaks and clonal relationship; but in our study we evaluated the VRE rate at ICUs not during an outbreak status. Effective infection control measures may be a factor for not having a high VRE prevalence. Many investigators worked on the detection of *vanA* or *vanB* from rectal samples or colonies by PCR. These methods have the advantage of giving rapid results compared to culture based methods. Isolation from samples can have the problem of inhibition.^17^^-^^19^

Werner G et al compared direct cultivation on a selective solid medium, polymerase chain reaction from an enrichment broth, and the BD GeneOhm™ VanR Assay for identification of vancomycin-resistant enterococci in screening specimens. They reported the performance of the assay and compared with culture-based methods and subsequent PCR analysis in two university hospitals with a different VRE prevalence. A total of 1786 samples were analyzed. The overall sensitivity was 93.1%; the specificity was 87.0% mainly due to false-positive *vanB* results.^20^

Usacheva EA et al reported the multicenter evaluation of the BD GeneOhm VanR assay for direct, rapid detection of vancomycin-resistant *Enterococcus* species in perianal and rectal specimens. They studied 1,027 perianal and rectal swab specimens from three geographically distinct US sites. Direct swab specimens were tested by the assay and compared with direct culture. The sensitivity, specificity, and positive and negative predictive values of the assay were 93.2%, 81.9%, 54.4%, and 98.1%. According to the authors the specificity was limited because of false-positive results in the *vanB* portion of the assay. Specificity with perianal swabs was significantly greater than with rectal swabs. They think that the assay is a simple, rapid, and acceptable method for screening for VRE in a variety of populations in which *vanA* is the predominant genotype. Samples positive for the *vanB* genotype should be confirmed by culture owing to the apparent high number of false-positive results.^21^

In our study 43 *Enterococcus* *spp.* isolates were obtained from 135 perianal swab samples; but only two (3.6%) samples taken from the newborn ICU, had vancomycin resistance (*vanA* type) that was detected both by the BD GeneOhm™ VanR assay and culture. BD GeneOhm VanR assay detected *vanA* type resistance in sixteen samples (11.8%), *vanB* type resistance in four samples (2.9%) and one sample (0.7%) had both *vanA* and *vanB* types of vancomycin resistance. Only two *E. faecium* isolates isolated at newborn ICU were resistant to vancomycin and teicoplanin. The most common underlying diseases at adult ICU were pneumoniae (16.2%), cerebrovascular diseases (13.7%) and 31.2% of them had multiple underlying diseases. In adult ICU most of the patients had multiple underlying diseases and that may be an additional factor although none of the patients were confirmed by culture based techniques. The newborns had prematurity (70.9%), respiratuary distress syndrome (49.1%), transient tachypneae (7.5%). These underlying disorders in newborn ICU could have attributed to colonisation of VRE. Also two VRE positive babies could be affected by VRE carrier state of their mothers and besides they could have been transferred from another center already being a VRE carrier. Risk factors such as renal failure, GIS operation, diabetes mellitus, malignity and corticosteroid treatment could also have contributed to VRE colonisation detected by only molecular method in both of the ICUs. 

The sensitivity of the assay was 100% due to lack of false negative results; and the specifity was 85.7%. The positive predictive value (PPV) was 9.5% and the negative predictive value (NPV) was 100%. The PPV mainly depends on the prevalance of that population and that may be the cause of low PPV. The low prevalance in our study group may be due to effective infection control measures; although the population that we have screened had underlying disorders, treated with broad spectrum antibiotics and hospitalized at ICUs. We also had discordant results such as simultaneous PCR positivity and culture negativity that can be due to dead enterococci. We had more PCR positivity than culture positivity. This can be due to poor culture growth, inhibition, contamination or not having enough inoculum for bacterial growth. Although molecular methods have the advantage of rapidity and sensitivity; they also have some disadvantages like high cost, DNA extraction problems, detecting DNA although the bacteria being dead, contamination, inhibition and false positivity.^7^^,^^22^^,^^23^

As a conclusion culture based methods require at least 48–72 hours; but PCR assays result is available in two to three hours. The most important disadvantage of these molecular assays is inhibition due to feacal samples and contamination. Against all, molecular methods decreased hospital stay and cost. GeneOhm VanR assays can be used as VRE screening tests in populations that show a predominant colonization with *vanA* containing enterococci, while *vanB* positive samples need to be confirmed by another method for the existence of VRE. In our study we detected more VRE colonisation by PCR compared to culture which may be due to detecting bacteria already being dead in PCR, poor culture growth or not having enough for bacterial growth. Early detection of VRE is an important issue especially in ICUs and molecular techniques are important tools; but we still need to confirm this method with culture based techniques and in order to do this further studies with higher number of patients with VRE colonisation are required.

## Authors Contribution

All the authors have contributed to the manuscript. The first four authors have contributed both to the writing of the manuscript and recording the data of the study. The fifth author have also contributed to the collection and recording of the data.

## References

[1] Bender EA, Peixoto de Freitas AL, Reiter KC, Lutz L, Barth AL (2009). Identification antimicrobial resistance and genotypic characterization of enterococcus spp isolated in Porto Alegre, Brazil. Brazilian J Microbiol.

[2] Neely AN, Maley MP (2000). Survival of enterococci and staphylococci on hospital fabrics and plastic. J Clin Microbiol.

[3] Tendolkar PM, Baghdayan AS, Shankar N (2003). Pathogenic enterococci: new developments in the 21st century. Cell Mol Life Sci.

[4] Leclerq R, Derlot E, Duval J, Courvalin P (1988). Plasmid-mediated resistance to vancomycin and teicoplanin in Enterococcus faecium. N Engl J Med.

[5] Diekema, DJ, Dodgson KJ, Sigurdardottir B, Pfaller M (2004). Rapid detection of antimicrobial-resistant organism carriage: an unmet clinical need. J Clin Microbiol.

[6] Diekema DJ, Edmond MB (2007). Look before you leap: active surveillance for multidrug resistant organisms. Clin Infect Dis.

[7] Malhotra-Kumar S, Haccuria K, Michiels M, Ieven M, Poyart C, Hryniewicz W (2008). Current trends in rapid diagnostics for methicillin-resistant Staphylococcus aureus and glycopeptide-resistant Enterococcus species. J Clin Microbiol.

[8] Clinical and Laboratory Standards Institute (2005). Performance standards for antimicrobial susceptibility testing: 15th informational supplement M100-S15.

[10] Chlebicki MP, Kurup A (2008). Vancomycin resistant enterococcus – a review from a Singapore perspective. Ann Acad Med Singapore.

[11] Patel R (2003). Clinical impact of vancomycin-resistant enterococci. J Antimicrob Chemother.

[12] Novicki TJ, Schapiro JM, Ulness BK, Sebeste A, Busse-Johnston L, Swanson KM (2004). Convenient selective differential broth for isolation of vancomycin-resistant Enterococcus from fecal material. J Clin Microbiol.

[13] Akinci E, Balik I, Tekeli E (1999). Antimicrobial Susceptibility of Enterococcus Species Collected from Clinical Specimens. Flora.

[14] Tufan ZK, Arslan S, Cesur S, Irmak H, Kinikli S (2010). Absence of vancomycin-resistant enterococci (VRE) despite the presence of risk factors: a survey of rectal carriage of VRE. Turk J Med Sci.

[15] Yis R, Aslan S, Citak C, Degirmenci S (2011). Evaluation of Vancomycin-Resistant Enterococcus Colonization at Gaziantep Children’s Hospital, Turkey. Mikrobiyol Bul.

[16] Zer Y, Bayram A, Akin Ozgur E, Balci I (2011). Analysis of the molecular epidemiology and antibiotic sensitivity of vancomycin resistant enterococci. Gaziantep Med J.

[17] Palladino S, Kay ID, Costa AM (2003). Real time PCR for the rapid detection of vanA and vanB genes. Diagn Microbiol Infect Dis.

[18] Palladino S, Kay ID, Flexman JP (2003). Rapid detection of vanA and vanB genes directly from clinical specimens and enrichment broths by real-time multiplex PCR assay. J Clin Microbiol.

[19] Satake S, Clark N, Rimland D, Nolte FS, Tenover FC (1997). Detection of vancomycin-resistant enterococci in fecal samples by PCR. J Clin Microbiol.

[20] Werner G, Serr A, Schutt S, Schneider C, Klare I, Witte W (2011). Comparison of direct cultivation on a selective solid medium, polymerase chain reaction from an enrichment broth, and the BD GeneOhm™ VanR Assay for identification of vancomycin-resistant enterococci in screening specimens. Diagn Microbiol Infect Dis.

[21] Usacheva EA, Ginocchio CC, Morgan M, Maglanoc G, Mehta MS, Tremblay S (2010). Prospective, multicenter evaluation of the BD GeneOhm VanR assay for direct, rapid detection of vancomycin-resistant Enterococcus species in perianal and rectal specimens. Am J Clin Pathol.

[22] Francois P, Bento M, Renzi G, Harbarth S, Pittet D, Schrenzel J (2007). Evaluation of three molecular assays for rapid identification of methicillin-resistant Staphylococcusaureus. J Clin Microbio.

[23] Gulay Z (2009). Rapid Diagnostic Methods for the Control of Multi-Drug Resistant Nosocomial Microorganisms. Ankem.

